# Editorial: HLA-G-Mediated Immune Tolerance: Past and New Outlooks

**DOI:** 10.3389/fimmu.2016.00653

**Published:** 2016-12-27

**Authors:** Silvia Gregori

**Affiliations:** ^1^Division of Regenerative Medicine, San Raffaele Telethon Institute for Gene Therapy (SR-Tiget), IRCCS San Raffaele Scientific Institute, Milan, Italy

**Keywords:** HLA antigens, tolerance, pregnancy, infection, autoimmune diseases, genetic variation

**Editorial on the Research Topic**

**HLA-G-Mediated Immune Tolerance: Past and New Outlooks**

This research topic gathered several researchers who actively base their research and investigations on the non-classical HLA-G molecule. HLA-G differs from classical HLA class I molecules since it has limited protein variability, can be expressed in several membrane-bound and soluble isoforms generated by alternative splicing generating, and modulates immune response. HLA-G expression is physiologically restricted to the maternal–fetal interface and to immune privileged adult tissues. *De novo* expression of HLA-G is deleterious when present in tumor cells and in chronically infected cells, whereas it is advantageous in autoimmune diseases and after transplantation.

In the present collection of manuscripts, different aspects of the HLA-G biology have been discussed including the genetic variability, the relationship between HLA-G and other non-classical HLA class I molecules, and the role of HLA-G in promoting tolerance in T-cell-mediated diseases and in pregnancy.

In the contest of HLA-G genetics, it is intriguing that the overall *HLA-G* gene structure was preserved during the evolution, and the HLA-G variability has been established before human dispersion from Africa. Castelli et al. illustrated that most of the variation sites found in the HLA-G coding region are either synonymous or intronic mutations and that the HLA-G promoter region presents numerous polymorphic sites.

Regarding the role of HLA-G in maintaining tolerance, Rizzo et al. and Dias et al. delivered a solid snap shot on the physiological expression of HLA-G and its role in inducing tolerance in autoimmunity. The Authors also discussed that the *de novo* expression of HLA-G, specifically in tumors and after chronic infections, has important implication in promoting immune escape. Special attention received the association between HLA-G polymorphisms, specifically those present at 3′UTR of the gene, protein expression, and functions in healthy and pathological conditions.

HLA-G belongs to the HLA class Ib molecules family that contains HLA-E and HLA-F. Morandi and Pistoia summarized, for the first time, the relationship between HLA-G and HLA-E in different settings. They concluded that, in physiological conditions, HLA-E expression is strongly associated with HLA-G and both molecules co-operate in promoting anergy in immune effector cells, specifically in NK cells. Conversely, HLA-G/HLA-E interaction in pathological conditions, i.e., in autoimmune and inflammatory diseases, may exert divergent or potentially opposite effects. The central role mediated by the non-classical HLA class I molecules, HLA-G, HLA-E, and HLA-F, in promoting tolerance during pregnancy and preeclampsia has been extensively discussed. Djurisic and Hviid indicated that in preeclampsia, HLA-F function is still unknown and that despite HLA-E is involved in immune suppression, increased soluble HLA-E levels has not been associated with preeclampsia. Conversely, the high expression of HLA-G compared to HLA-E and -F in the placenta, and the presence of HLA-G in semen, endometrium, in matured cumulus–oocyte complex, as well as the rise in soluble level after conception, imply an important role for HLA-G in early pregnancy. Furthermore, the role of HLA-G in immune regulation and spiral artery remodeling highlights its importance and multifaceted activities. In line with this view, Gregori et al. proposed that, at the fetal/maternal interface, the expression of HLA-G coordinates the cross-talk between fetal extravillous trophoblasts (EVTs) and maternal decidual and immune cells. Upon blastocyst is implantation into the uterine wall, trophoblasts indeed differentiate into EVTs that regulate their cell migration in the decidua, support the induction of the pro-angiogenic microenvironment necessary for vascular remodeling, inhibit effector innate and adaptive immune responses, and promote a tolerogenic loop in which resident cells become tolerogenic.

Finally, Rebmann et al. presented and discussed a new and interesting novel aspect in the biology of the HLA-G, the HLA-G-bearing extracellular vesicles (EVs). Several cell types involved in immune tolerance and tissue remodeling, including tumor cells, trophoblasts, and mesenchymal stromal cells, secrete HLA-G-bearing EVs. The mechanisms underlying the functional consequences of HLA-G-bearing EVs are, thus far, little investigated. Nevertheless, HLA-G-bearing EVs represent a novel mode of HLA-G delivery within target cells, thereby bypassing the interaction between HLA-G and its specific receptors. This new concept opens new perspectives in the modulatory activity of HLA-G.

Overall, we gathered a nice compilation of old and new findings on HLA-G, and this research topic highlights the importance of this immune-modulatory molecule in healthy and pathological conditions and proposes new investigation avenue to better define HLA-G biology and potentially identify new therapeutic strategies for promoting or dampening tolerance.

## Author Contributions

This work was supported by Telethon Italy grant number Tele 16-G1. The author confirms being the sole contributor of this work and approved it for publication.

## Conflict of Interest Statement

The author declares that the research was conducted in the absence of any commercial or financial relationships that could be construed as a potential conflict of interest.

